# Dynamic Sensorless Control Approach for Markovian Switching Systems Applied to PWM DC–DC Converters with Time-Delay and Partial Input Saturation

**DOI:** 10.3390/s23156936

**Published:** 2023-08-04

**Authors:** Abdelmalek Zahaf, Sofiane Bououden, Mohammed Chadli, Ilyes Boulkaibet, Bilel Neji, Nadhira Khezami

**Affiliations:** 1 Faculty of Technology Sciences, Constantine 1—Frères Mentouri University, Constantine 25017, Algeria; 2 Laboratory of SATIT, Department of Industrial Engineering, Abbes Laghrour University, Khenchela 40004, Algeria; sofiane.bououden@univ-khenchela.dz; 3 Université Paris-Saclay, Univ Evry, IBISC, 91020 Evry, France; mohammed.chadli@univ-evry.fr; 4 College of Engineering and Technology, American University of the Middle East, Egaila 54200, Kuwait; ilyes.boulkaibet@aum.edu.kw (I.B.); nadhira.khezami@aum.edu.kw (N.K.)

**Keywords:** PWM DCDC converters, Markovian switching system, partial input saturation, observer–predictive control, disturbance rejection

## Abstract

This paper provides a detailed analysis of the output voltage/current tracking control of a PWM DCDC converter that has been modeled as a Markov jump system. In order to achieve that, a dynamic sensorless strategy is proposed to perform active disturbance rejection control. As a convex optimization problem, a novel reformulation of the problem is provided to compute optimal control. Accordingly, necessary less conservative conditions are established via Linear Matrix Inequalities. First, a sensorless active disturbance rejection design is proposed. Then, to carry out the control process, a robust dynamic observer–predictive controller approach is introduced. Meanwhile, the PWM DC-DC switching power converters are examined as discrete-time Markovian switching systems. Considering that the system is subject to modeling uncertainties, time delays, and load variations as external disturbances, and by taking partial input saturation into account, the Lyapunov–Krasovskii function is used to construct the required feasibility frame and less conservative stability conditions. As a result, the proposed design provides an efficient control strategy with disturbance rejection and time-delay compensation capabilities and maintains robust performance with respect to constraints. Finally, a PWM DC-DC power converter simulation study is performed in different scenarios, and the obtained results are illustrated in detail to demonstrate the effectiveness of the proposed approach.

## 1. Introduction

Due to their efficiency and adaptability to various systems, DC-DC converters have gained significant attention in the industry. They have been used by many researchers as one of the nonlinear plants to test different nonlinear control strategies. Generally, DC-DC converters, such as buck type [1,2,3], boost type [4,5,6,7,8], and buck–boost type [9,10,11], are highly nonlinear systems due to the incorporated switch, which causes the process to change dramatically within one operating cycle. The objective of the switching control in DC-DC converters is to realize high power transfer efficiency and to ensure good output voltage tracking (maintaining the desired levels of the output voltage/current). In addition, DC-DC converters have been widely used in different applications for their usefulness and functionalities in the control process. These applications include robotics [12], networking, motor driving [13], and renewable energy [14,15,16,17]. Meanwhile, two aspects of DC-DC converters have been investigated over the years in the literature: the modeling aspect and control approaches. Inspired by their usefulness and applicability, this study focuses on DC-DC converters with PWM (Pulse-Width-Modulation). PWM had been applied to DC-DC switching converters in many applications, such as in [18], in which the result illustrated a significant improvement in voltage regulation.

Relating to switched-mode converters, DC-DC power systems have been used as one of the main benchmarks to study and control Markov jump systems (MJSs). MJSs have random abrupt changes in their parameters and structures. Therefore, the stability of discrete-time Markov jump linear systems is a very challenging criterion. Many researchers have investigated several techniques to improve the stability of these systems [19,20]. In addition, modeling these types of systems results in extra uncertain parameters. To cope with uncertainty issues, several works have considered the filtering task and implemented it as a pointed control design [21]. As is well known, DC-DC converters are extremely nonlinear plants. Usually, these classes of converters are affected by external factors such as load variation, which is considered an external disturbance and can result in a chattering influence at the output voltage/current response. Handling uncertainties and achieving disturbance rejection while controlling a system have been investigated in many control designs. With DC-DC converters, improving the control of output responses is necessary, especially for dealing with chattering alleviation resulting from disturbances and time delays.

To this subject, the sensorless disturbance rejection control strategy is considered to be among the most efficient control designs; it has been implemented to maintain the highest performance of DC-DC converters. Therefore, the filtering task and extended state observer “ESO” concepts have been used on a very large scale to enhance the controller design performance. In fact, for its accuracy of output estimation, observer-based control design has been widely employed in real-time processes and industrial applications. In order to study the estimation response of hybrid systems, an observer was tested in [22] to establish a controller, while an observer-based control design was proposed in [23] for switched affine systems applied to DC–DC converters. Furthermore, a mismatch in functional dynamics can have occurred from disturbances and time delays. To cope with undesirable situations, and to improve the reliability of the controller for linear systems subject to mixed delays and stochastic delays, delay-kernel-dependent and distributed-delay-dependent approaches were proposed in [24,25]. Meanwhile, diagnosis tasks may become very useful in this case, e.g., the reliable proposed diagnosis approach in [26], based on predictive control. To this end, time-delay-based predictive control was designed in [27] as a second-step control scheme, in order to maintain the very high performance of the DC-DC converters and ensure acceptable compensation for the time delays. Furthermore, in [28], an ESO-based sliding mode control design was introduced to handle disturbances.

In fact, sensorless control designs are more reliable and less time-consuming for electric and network applications. As a tracking current sensorless mode strategy, a finite-time output feedback control design based on a reduced-order observer was investigated in [29] to control a DC-DC buck-switched power converter. Recently, many studies have been devoted to sensorless control-based observers. A sensorless control design based on a state observer was given in [30] to estimate the unknown load conductance. Next, a generalized parameter estimation-based observer was introduced in [31] for a DC-DC boost converter. In [32], an investigation was performed for robust speed tracking control under network-induced delay and slope variation with state estimation; this study may be improved in the future and used in electric vehicles. Additionally, the employment of the estimation concept-based state observers fused with many approaches has been investigated many times and established as a sensorless control strategy. Sensorless adaptive voltage control for DC-DC converters was proposed in [33], and extended to studying the robustness of bidirectional DC-DC converters in [34]. In addition, buck converters are controlled using adaptive sensorless control with a constant power load [35], while a disturbance observer-based sliding mode control is proposed in [36], to enhance the dynamic performance of a buck/boost converter.

Despite the existing control strategies, predictive control has the ability to provide high robustness and high response time. In addition, model predictive control is a control design that can deal with terminal constraints. As a consequence, combining predictive control with an observer may result in a reliable sensorless predictive control strategy. Note that a current observer is usually chosen to build a sensorless predictive control approach for DC-DC converters. In order to decrease the effect of component parasitic parameters, the authors in [37] proposed a self-correction differential current observer approach. A sensorless model predictive control scheme is proposed in [38] to improve the dynamic performances of dual bridge DC-DC converters. Generally, improving the controller performances can be achieved by providing less conservative conditions, either in the presence of disturbances and time delay or during the transient response of the voltage/current control. This approach may reduce the calculation complexity of the provided control design and avoid consuming time in updating the controller’s parameters. Unfortunately, is not always easy to design such a technique for PWM DC-DC switching power converters, since the appearance of the threshold value of Diode as input saturation along time for DC-DC working mode “buck/boost mode”. Meanwhile, the partial input saturation is not matched in the PWM DC-DC converters model, which eventually increases the calculation complexity of new parameters of the controller.

Motivated by the previous proposed work based on predictive control, this work focuses on enhancing the sensorless disturbance rejection control strategy. By employing a model predictive control scheme based on extended state observer, efficient feasible, and stable conditions have been provided. The robust disturbance rejection for PWM DC-DC converters is a very sensitive criterion, as well as the compensation for time delays. Also, satisfying accurate output voltage/current tracking as terminal constraints is very essential in the proposed controller. Mainly, PWM DC-DC switching power converters are considered high nonlinear systems, usually modeled as discrete-time Markovian jump systems. In this work, a new reformulation has been given as a linear system with modeling uncertainties. The obtained augmented system reflects the discrete-time Markovian jump system that describes the DC-DC converter. Thus, a dynamic sensorless active disturbances rejection control approach is proposed. Accordingly, and using supported assumptions, less conservative conditions have been established via Linear Matrix Inequalities based on Lyapunov–Krasovskii function. While the asymptotical stability of the PWM DC-DC power converter is ensured satisfying all constraints. The main contributions of this work can be summarized in the next points:

- The disturbance rejection and chattering alleviation are achieved to maintain robust performances of PWM DC-DC converters. Mainly, the two-mode tracking control “voltage/current control” had been supported based on an extended state observer;
- Compensating the time delay is performed at each time sample (iteration). Additionally, the partial input saturation has been modeled as an explicit parameter in the state representation. As a required performance, the proposed approach stabilizes the behavior of the PWM DC-DC converters by satisfying the terminal constraints for the output voltage/current tracking;
- At the establishment of stability and feasibility conditions, a supported assumption is given to eliminate the bilinearity form so that, to update the controller parameters at each iteration, the infinite time domain “min–max” is implemented to formulate the optimization problem as a relaxed convex optimization problem.

This paper is presented as follows: Section 2 introduces the problem formulation and the model of DC-DC converters. Section 3 presents the design of the sensorless active disturbance rejection approach based on an observer to estimate and stabilize the behavior of Markovian switching systems. In Section 4, we introduce an efficient robust dynamic controller. Therefore, necessary and stable conditions are constructed using a robust observer–predictive control design. We conclude the paper with Section 5, PWM DC-DC converter as a demonstrated case study is presented with simulation results for two scenarios.

Notation: Throughout this paper, the symbols are quite standard unless otherwise specified. $R^{n}$ denotes the n dimensional Euclidean space and $S^{n\times n}{,R}^{n\times m},R^{m\times n},$ are the set of n×n, n×m, and m×n real matrices, respectively. $0_{n}$ and I represent the zero and identity matrices with proper dimensions, respectively. *K*_1_ and *K*_2_ are the controller gains matrices, *L* is the observer gain matrix and $d_{k}$ is a varying delay.

*Q, G,* $\bar{P}$*,*${\bar{Q}}_{i,\;i=1:2}$ and $X_{i,\;i=1:2}$ symmetric positive definite matrices.${\;Q}_{0}\;and\;R_{0}$ are weighting matrices and γ positive scalar. The symbol * denotes the elements above the main diagonal of a symmetric block matrix.

## 2. Problem Formulation and Modelling of DC-DC Converters

In this paper, we focus on analyzing Pulse Width Modulation (PWM) converters, which had analyzed in different ways in the literature by applying averaging method. A nonlinear state-space representation of the DC-DC converter can be expressed in the following form as [18]:

First, the mode on-state of the MOSFET transistor case for the DC-DC converter is given by:

(1) $$\begin{array}{cc} \left[\begin{array}{c} {\dot{I}}_{l}(t)\\ {\dot{V}}_{o}(t) \end{array}\right] & +E\left[\begin{array}{c} {\dot{I}}_{l}(t-d)\\ {\dot{V}}_{o}(t-d) \end{array}\right]\\ & \;\;\;\;\;\;\;\;\;\;\;\;\;\;\;\;\;=\left[\begin{array}{cc} \left(-1/L_{l}\right)\left(R_{l}+R_{p}\right) & {-R}_{p}/L_{l}R_{c}\\ R_{p}/C_{c}R_{c} & {-R}_{p}/C_{c}{RR}_{c} \end{array}\right]\left[\begin{array}{c} I_{l}(t)\\ V_{o}(t) \end{array}\right]\\ & \;\;\;\;\;\;\;\;\;\;\;\;\;\;\;\;\;+\left[\begin{array}{c} (V_{in}+(R_{m}I_{l}(t)))/L_{l}\\ 0 \end{array}\right]+\left[\begin{array}{c} R_{p}/L_{l}\\ -R_{p}/(C_{c}R_{c}) \end{array}\right]I_{load}(t)\;. \end{array}$$

In the second mode, the model is given in the off-state of the MOSFET transistor case by the next equation:

(2) $$\begin{array}{cc} \left[\begin{array}{c} {\dot{I}}_{l}(t)\\ {\dot{V}}_{o}(t) \end{array}\right] & +E\left[\begin{array}{c} {\dot{I}}_{l}(t-d)\\ {\dot{V}}_{o}(t-d) \end{array}\right]\\ & \;\;\;\;\;\;\;\;\;\;\;\;\;\;\;\;\;=\left[\begin{array}{cc} \left(-1/L_{l}\right)\left(R_{l}+R_{p}\right) & {-R}_{p}/L_{l}R_{c}\\ R_{p}/C_{c}R_{c} & {-R}_{p}/C_{c}{RR}_{c} \end{array}\right]\left[\begin{array}{c} I_{l}(t)\\ V_{o}(t) \end{array}\right]+\left[\begin{array}{c} -V_{d}/L_{l}\\ 0 \end{array}\right]\\ & \;\;\;\;\;\;\;\;\;\;\;\;\;\;\;\;\;+\left[\begin{array}{c} R_{p}/L_{l}\\ -R_{p}/(C_{c}R_{c}) \end{array}\right]I_{load}(t)\; \end{array}$$

where the global dynamic model of the converters is presented in [11] as:

(3) $$\begin{array}{cc} \left[\begin{array}{c} {\dot{I}}_{l}(t)\\ {\dot{V}}_{o}(t) \end{array}\right] & +E\left[\begin{array}{c} {\dot{I}}_{l}(t-d)\\ {\dot{V}}_{o}(t-d) \end{array}\right]\\ & \;\;\;\;\;\;\;\;\;\;\;\;\;\;\;\;\;=\left[\begin{array}{cc} \left(-1/L_{l}\right)\left(R_{l}+R_{p}\right) & {-R}_{p}/L_{l}R_{c}\\ R_{p}/C_{c}R_{c} & {-R}_{p}/C_{c}{RR}_{c} \end{array}\right]\left[\begin{array}{c} I_{l}(t)\\ V_{o}(t) \end{array}\right]+\left[\begin{array}{c} -V_{d}/L_{l}\\ 0 \end{array}\right]\\ & \;\;\;\;\;\;\;\;\;\;\;\;\;\;\;\;\;+\left[\begin{array}{c} \frac{V_{in}+V_{d}+\left(R_{m}I_{l}\left(t\right)\right)}{L_{l}}\\ 0 \end{array}\right]d(t)+\left[\begin{array}{c} R_{p}/L_{l}\\ -R_{p}/(C_{c}R_{c}) \end{array}\right]I_{load}(t)\; \end{array}$$

where $I_{l}(t)$ and $V_{o}(t)$ are the inductance current and output voltage of the PWM converter, respectively. $R_{m}$ is the on-state resistance of the MOSFET transistor, $R_{L}$ is the winding resistance of the inductor, $R_{p}=R∥R_{c}$ is the equivalent series resistance (while $R_{c}$ is the equivalent series resistance of the filter capacitor), $V_{d}$ is the threshold voltage of the diode and $i_{load}(t)$ represent the external disturbance. Finally, d(t) is the duty cycle.

**DC-DC Converters Model:**

First, we define:

(4) $$\dot{z}\left(t\right)={\left[{\dot{I}}_{l}\left(t\right)\;{\dot{V}}_{o}(t)\;\right]}^{T}$$

(5) $$\begin{array}{cc} \dot{z}\left(t\right)+E\dot{z}\left(t-d\right) & =\left[\begin{array}{cc} \left(-1/L_{l}\right)\left(R_{l}+R_{p}\right) & {-R}_{p}/L_{l}R_{c}\\ R_{p}/C_{c}R_{c} & {-R}_{p}/C_{c}{RR}_{c} \end{array}\right]z\left(t\right)+\left[\begin{array}{c} -\frac{V_{d}}{L_{l}}\\ 0 \end{array}\right]+\left[\begin{array}{c} \frac{V_{d}}{L_{l}}\\ 0 \end{array}\right]d\left(t\right)+\left[\begin{array}{c} R_{m}z\left(t\right)/L_{l}\\ 0 \end{array}\right]d(t)\\ & +\left[\begin{array}{c} V_{in}/L_{l}\\ 0 \end{array}\right]d(t)+\left[\begin{array}{c} R_{p}/L_{l}\\ -R_{p}/(C_{c}R_{c}) \end{array}\right]I_{load}(t)\; \end{array}$$

(6) $$\dot{z}\left(t\right)+E\dot{z}\left(t-d\right)=Az\left(t\right)+u(t)+DE(t)$$

where A, D, and E are represented the matrix state, disturbances matrix, and matrix function of the Markov process. u(t) represents input control.

Where (6) can be written as follows:

(7) $$\bar{E}\dot{\Psi }\left(t\right)=\bar{A}\Psi \left(t\right)+u(t)+DE(t)$$

For a general state space representation of (7) we obtain:

(8) $$\dot{\Psi }\left(t\right)=\tilde{A}\Psi \left(t\right)+\tilde{B}u(t_{k})+\tilde{D}E(t)\;$$

The defining matrices of (7) and (8) are next:

$\tilde{A}={\bar{E}}^{-1}\bar{A}$, $\tilde{B}={\bar{E}}^{-1}$ and $\tilde{D}={\bar{E}}^{-1}D$

$\bar{E}=\left[\begin{array}{cc} 0 & E \end{array}\right]$, $\bar{A}=\left[\begin{array}{cc} A & 0 \end{array}\right]$ and $E_{1}=\left[\begin{array}{cc} 1 & 0 \end{array}\right]$,

$$A=\left[\begin{array}{cc} \left(-1/L_{l}\right)\left(R_{l}+R_{p}\right) & {-R}_{p}/L_{l}R_{c}\\ R_{p}/C_{c}R_{c} & {-R}_{p}/C_{c}{RR}_{c} \end{array}\right],D=\left[\begin{array}{c} R_{p}/L_{l}\\ -R_{p}/(C_{c}R_{c}) \end{array}\right]$$

(9) $$u\left(t\right)=\left[\begin{array}{c} V_{in}/L_{l}\\ 0 \end{array}\right]d(t)+\left[\begin{array}{c} -V_{d}/L_{l}\\ 0 \end{array}\right]+\left[\begin{array}{c} V_{d}/L_{l}\\ 0 \end{array}\right]d(t)+\left[\begin{array}{c} R_{m}E_{1}\Psi \left(t\right)/L_{l}\\ 0 \end{array}\right]d(t)$$

(10) $$u\left(t\right)=u_{sat}\left(t\right)+u_{c}\left(t\right)$$

(11) $$\left\{\begin{array}{c} u_{c}\left(t\right)=\left[\begin{array}{c} R_{m}E_{1}/L_{l}\\ 0 \end{array}\right]d\left(t\right)\Psi \left(t\right)\;\\ u_{sat}\left(t\right)=\left[\begin{array}{c} V_{in}/L_{l}\\ 0 \end{array}\right]d\left(t\right)+\left[\begin{array}{c} V_{d}/L_{l}\\ 0 \end{array}\right]\left(d\left(t\right)-1\right)\; \end{array}\right.$$

In this paper, a discrete uncertain linear system is investigated, so, that the sampling time is 0.095 ms and the PWM frequency used in this work equals 1 kHz.

As a result, we consider the following discrete-time uncertain switching system with disturbances and time delay that reflected the system (8) represented as:

(12) $$\left\{\begin{array}{c} \Psi \left(k+1\right)=A\Psi \left(k\right)+Bu(k)+DE(k)\\ Y_{\Psi }\left(k\right)=C\Psi \left(k\right)\; \end{array}\right.\;$$

While the controller is required to satisfy the output constraints as follows:

(13) $${\left\|Y_{\Psi }\left(k+i/k\right)\right\|}_{max}={\left\|Y_{\Psi }\left(k\right)\right\|}_{2}\leq y_{max}^{2}.$$

Then, the expression (11) can be written as follows:

(14) $$\left\{\begin{array}{c} u\left(k\right)=u_{sat}\left(k\right)+u_{c}\left(k\right)\;\\ u_{c}\left(k\right)=K\hat{\Psi }\left(k\right)\; \end{array}\;\right..$$

**Remark** **1.** *To create a conducting path along the diode, a minimum voltage value “the threshold voltage of the diode” is needed. Since a real diode is never completely off, the achievement of the threshold value is not always accurate at a specific time. So, by controlling and increasing the voltage applied to the diode, the required threshold voltage value is obtained. As a result, the time delay in this paper is considered in the input control.*

**Assumption** **1.** *The threshold voltage value is considered as a partial input saturation since the threshold voltage is known as the point where the diode starts conducting in the exponential mode.*

Combined with Remark 1 and the input control (10), with respect to time along the trajectory for the operating mode of power converters, the global control law is expressed as:

(15) $$u\left(k\right)=\left\{\begin{array}{c} u\left(k-d\right)=u_{sat}\left(k-d\right)+u_{c}\left(k\right)\;v_{d}<V_{d}\;\\ u\left(k\right)=u_{sat}\left(k\right)+u_{c}\left(k\right)\;v_{d}=V_{d}\; \end{array}.\right.$$

For any instant $\left\{k/k\right\}$ along the trajectory of the system, and taking into account the duty cycle $d\left(t\right)$ of the input control law, the plant system can be expressed as follows:

(16) $$\Psi \left(k+1\right)=A\Psi \left(k\right)+Bu\left(k\right)+Bu\left(k-d\right)+DE(k)$$

In this work, it is assumed that the states of the discrete-time uncertain switching system with disturbances and time delay are not measurable. So, an observer is designed taking into account input saturation:

(17) $$\left\{\begin{array}{c} \hat{\Psi }\left(k+1\right)=A\hat{\Psi }\left(k\right)+Bu\left(k\right)+Bu\left(k-d\right)+D\hat{E}\left(k\right)+L\left(y\left(k\right)-\hat{y}\left(k\right)\right)\\ {\hat{Y}}_{\Psi }\left(k\right)=C\hat{\Psi }\left(k\right)\;\\ e_{E}\left(k+1\right)={℘}_{e}\left(Y_{\Psi }\left(k\right)-{\hat{Y}}_{\Psi }\left(k\right)\right)\; \end{array}\right.\;$$

Then, the closed loop linear plant can have written as:

(18) $$\left\{\begin{array}{c} \hat{\Psi }\left(k+1\right)=A\hat{\Psi }\left(k\right)+Bu\left(k\right)+Bu\left(k-d\right)+D\hat{E}\left(k\right)+LCe\left(k\right)\\ {\hat{Y}}_{\Psi }\left(k\right)=C\hat{\Psi }\left(k\right)\;\\ e_{E}\left(k+1\right)={℘}_{e}Ce\left(k\right)\; \end{array}\right.$$

where A,B, and C are state matrices, $K_{i}$ are the controller gains matrix, and *L* is the observer gain matrix, where D and ${℘}_{e}$ are disturbance matrices, respectively.

For necessary stability conditions, a Lyapunov–Krasovskii function is defined as:

(19) $$\begin{array}{cc} V\left(k/k\right)= & \underset{V_{1}}{\underbrace{{\hat{\Psi }}^{T}\left(k\right)P\hat{\Psi }\left(k\right)}}+\underset{V_{2}}{\underbrace{\sum_{j=k-d_{k}}^{k-1}\eta^{T}\left(j\right)Q_{1}\eta \left(j\right)}+}\\ & \;\;\;\;\;\;\;\;\;\;+\underset{V_{3}}{\underbrace{\sum_{j=1-d_{M}}^{1-d_{m}}\sum_{r=k-1-j}^{k-1}\eta^{T}\left(r\right)Q_{2}\eta \left(r\right)}} \end{array}$$

**Assumption** **2** ([12]). *Let* X* and* Y* be known matrices with appropriate dimensions. The following statements are equivalent and verified for any* $P=P^{T}>0\;$*if and only if there exists an appropriate matrix* Z*, such that:*

(20) $$1.\;\;\;\;\;X^{T}PY+Y^{T}PX\leq 0$$

(21) $$2.\;\;Z{+X}^{T}PX+Y^{T}PY\leq 0.$$

## 3. Sensorless Active Disturbance Rejection Design

Based on estimated load variation information by sensorless active disturbance rejection-based extended state observer, the controller can react to the disturbance rapidly and compensate for the time delay and would have improved dynamic performance. So, the next theorem is given:

**Theorem** **1.** *For the discrete-time Markovian switching systems (12). The state observer (17) meets the control performances (15) and will asymptotically stabilize the system (5), subject to disturbance, time delay, and partial input saturation if there exists a positive definite matrices* $\bar{P},\;{\bar{Q}}_{1}\;and\;{\bar{Q}}_{2}$ *; and any matrices* $K_{i},\;L$ *and* $Z_{i}$ *satisfying the following LMI:*

(22) $$\left[\begin{array}{ccccccccccc} \xi \left(\bar{Z}\right) & * & * & * & * & * & * & * & * & * & *\\ \sqrt{2}\left(A+BK_{1}\right) & \bar{P} & * & * & * & * & * & * & * & * & *\\ -\omega_{1}\omega_{1}^{T} & 0_{n} & \bar{P} & * & * & * & * & * & * & * & *\\ 0_{n} & \sqrt{2}\left(BK_{2}\right) & 0_{n} & \bar{P} & * & * & * & * & * & * & *\\ 0_{n} & 0_{n} & \sqrt{2}D & 0_{n} & \bar{P} & * & * & * & * & * & *\\ 0_{n} & 0_{n} & 0_{n} & \sqrt{2}\left(LC\right) & 0_{n} & \bar{P} & * & * & * & * & *\\ 0_{n} & 0_{n} & 0_{n} & 0_{n} & \omega_{1} & 0_{n} & {\bar{Q}}_{1} & * & * & * & *\\ 0_{n} & 0_{n} & 0_{n} & 0_{n} & {-\omega }_{2} & 0_{n} & 0_{n} & {\bar{Q}}_{1} & * & * & *\\ 0_{n} & 0_{n} & 0_{n} & 0_{n} & 0_{n} & 0_{n} & 0_{n} & \sqrt{\left(d_{k}+1\right)}\omega_{1} & {\bar{Q}}_{2} & * & *\\ 0_{n} & 0_{n} & 0_{n} & 0_{n} & 0_{n} & 0_{n} & 0_{n} & {-\omega }_{3} & 0_{n} & {\bar{Q}}_{2} & *\\ 0_{n} & 0_{n} & 0_{n} & 0_{n} & 0_{n} & 0_{n} & 0_{n} & {-\omega }_{4} & 0_{n} & 0_{n} & {\bar{Q}}_{2} \end{array}\right]\geq 0\;$$

**Proof of Theorem** **1.** Taking the forward difference of (19) as $∆V\left(k\right)=V\left(k+1\right)-V\left(k\right)$ and with respect to time along the trajectory of the system yields:

(23) $$∆V\left(k\right)=V_{1}\left(k\right)+V_{2}\left(k\right)+V_{3}\left(k\right)$$

Define $∆V\left(k/k\right).$ as follows next:

(24) $$V(\hat{\Psi }(k+j+1/k))-V(\hat{\Psi }(k+j/k))\leq 0.$$

According to (17), that can be written as:

(25) $$\begin{array}{cc} {\left(A\hat{\Psi }\left(k\right)+Bu\left(k\right)+Bu\left(k-d\right)+D\hat{E}\left(k\right)+LCe\left(k\right)\right)}^{T}P(A\hat{\Psi }\left(k\right)+Bu\left(k\right)+Bu\left(k-d\right) & \\ \;\;\;\;\;\;\;\;\;\;\;\;\;\;\;\;\;\;\;\;+D\hat{E}\left(k\right)+LCe\left(k\right))-\left({\hat{\Psi }}^{T}\left(k/k\right)P\hat{\Psi }(k/k)\right)+\sum_{j=k-d_{k}}^{k-1}\eta^{T}\left(j\right)Q_{1}\eta \left(j\right) & \\ \;\;\;\;\;\;\;\;\;\;\;\;\;\;\;\;\;\;\;\;\;\;\;\;\;\;\;\;\;\;+\sum_{j=1-d_{M}}^{1-d_{m}}\sum_{r=k-1-j}^{k-1}\eta^{T}\left(r\right)Q_{2}\eta \left(r\right)\leq 0. & \end{array}$$

The previous Equation (25) is expressed as follows next:

(26) $$\begin{array}{cc} {\left((A+BK_{1})\hat{\Psi }\left(k\right)+BK_{2}\hat{\Psi }\left(k-d\right)+D\hat{E}\left(k\right)+LCe\left(k\right)\right)}^{T}P((A+BK_{1})\hat{\Psi }\left(k\right) & \\ \;\;\;\;\;\;\;\;\;\;\;\;\;\;\;\;\;\;\;\;\;\;\;\;\;\;\;\;\;\;\;\;\;\;\;\;\;\;\;\;\;\;\;\;\;\;\;\;\;+BK_{2}\hat{\Psi }\left(k-d\right)+D\hat{E}\left(k\right)+LCe\left(k\right))-\left({\hat{\Psi }}^{T}\left(k/k\right)P\hat{\Psi }(k/k)\right) & \\ \;\;\;\;\;\;\;\;\;\;\;\;\;\;\;\;\;\;\;\;\;\;\;\;\;\;\;\;\;\;\;\;\;\;\;\;\;\;\;\;\;\;\;\;\;\;\;\;\;+\eta^{T}\left(k\right)Q_{1}\eta \left(k\right)-\eta^{T}\left(k-d_{k}\right)Q_{1}\eta \left(k-d_{k}\right)+{\left(d_{k}+1\right)\eta }^{T}\left(k\right)Q_{2}\eta \left(k\right) & \\ \;\;\;\;\;\;\;\;\;\;\;\;\;\;\;\;\;\;\;\;\;\;\;\;\;\;\;\;\;\;\;\;\;\;\;\;\;\;\;\;\;\;\;\;\;\;\;\;\;-\eta^{T}\left(k-d_{M}\right)Q_{2}\eta \left(k-d_{M}\right)-\eta^{T}\left(k-d_{m}\right)Q_{2}\eta \left(k-d_{m}\right)\leq 0. & \end{array}$$

The next vectors and matrices are defined as:

(27) $$\eta \left(k\right)=\left[\begin{array}{cccc} \hat{\Psi }\left(k\right) & \hat{\Psi }\left(k-d\right) & \hat{E}\left(k\right) & e\left(k\right) \end{array}\right].$$

Then, it can be written as:

(28) $$\begin{array}{cc} {\left({\left[\begin{array}{ccc} \left(A+BK_{1}\right) & \begin{array}{cc} BK_{2} & D \end{array} & LC \end{array}\right]}^{T}\right)}^{T}\eta^{T}\left(k\right)\bar{P}\eta \left(k\right)\left({\left[\begin{array}{ccc} \left(A+BK_{1}\right) & \begin{array}{cc} BK_{2} & D \end{array} & LC \end{array}\right]}^{T}\right) & \\ \;\;\;\;\;\;\;\;\;\;\;\;\;\;\;\;\;\;\;\;\;\;\;\;\;\;\;\;-\left(\omega_{1}\eta^{T}\left(k\right)P\eta \left(k\right){\omega_{1}}^{T}\right)+\eta^{T}\left(k\right)Q_{1}\eta \left(k\right) & \\ \;\;\;\;\;\;\;\;\;\;\;\;\;\;\;\;\;\;\;\;\;\;\;\;\;\;\;\;-\eta^{T}\left(k-d_{k}\right)Q_{1}\eta \left(k-d_{k}\right)+{\left(d_{k}+1\right)\eta }^{T}\left(k\right)Q_{2}\eta \left(k\right) & \\ \;\;\;\;\;\;\;\;\;\;\;\;\;\;\;\;\;\;\;\;\;\;\;\;\;\;\;\;-\eta^{T}\left(k-d_{M}\right)Q_{2}\eta \left(k-d_{M}\right)-\eta^{T}\left(k-d_{m}\right)Q_{2}\eta \left(k-d_{m}\right)\leq 0\; & \end{array}$$

where $\bar{P}=\left[\begin{array}{ccc} P & P & P\\ P & P & P\\ P & P & P \end{array}\right]$ and $\omega_{i}\in R^{n\times n}\left(i=1,\ldots ,4\right),e.g.,\omega_{1}=\left[\begin{array}{cccc} I_{n} & 0_{n} & 0_{n} & 0_{n} \end{array}\right]$

(29) $$\begin{array}{cc} {\left({\left[\begin{array}{ccc} \left(A+BK_{1}\right) & \begin{array}{cc} BK_{2} & D \end{array} & LC \end{array}\right]}^{T}\right)}^{T}\omega_{1}^{T}\left({\bar{P}}_{1}+{\bar{P}}_{2}\right)\omega_{1}\left({\left[\begin{array}{ccc} \left(A+BK_{1}\right) & \begin{array}{cc} BK_{2} & D \end{array} & LC \end{array}\right]}^{T}\right) & \\ \;\;\;\;\;\;\;\;\;\;\;\;\;\;\;\;\;\;\;\;\;\;\;\;\;\;\;\;-\left(\omega_{1}\omega_{1}^{T}P\omega_{1}\omega_{1}^{T}\right)+\omega_{1}^{T}Q_{1}\omega_{1}-\omega_{2}^{T}Q_{1}\omega_{2}+\left(d_{k}+1\right)\omega_{1}^{T}Q_{2}\omega_{1} & \\ \;\;\;\;\;\;\;\;\;\;\;\;\;\;\;\;\;\;\;\;\;\;\;\;\;\;\;\;-\omega_{3}^{T}Q_{2}\omega_{4}-\omega_{4}^{T}Q_{2}\omega_{4}\leq 0\; & \end{array}$$

where:

(30) $$\zeta \left(k\right)={\left[\begin{array}{ccc} \eta^{T}\left(k\right) & \eta^{T}\left(k-d_{k}\right) & \begin{array}{cc} \eta^{T}\left(k-d_{M}\right) & \eta^{T}\left(k-d_{m}\right) \end{array} \end{array}\right]}^{T}\;$$

${\bar{P}}_{1}=\left[\begin{array}{ccc} P & 0_{n} & 0_{n}\\ 0_{n} & P & 0_{n}\\ 0_{n} & 0_{n} & P \end{array}\right]$, and ${\bar{P}}_{2}=\left[\begin{array}{ccc} 0_{n} & P & P\\ P & 0_{n} & P\\ P & P & 0_{n} \end{array}\right]$.

Using Assumption 2, the inequality (30) leads to:

(31) $$\begin{array}{cc} 2{\left(A+BK_{1}\right)}^{T}P\left(A+BK_{1}\right)+2{\left(BK_{2}\right)}^{T}P\left(BK_{2}\right)+2D^{T}PD+2{\left(LC\right)}^{T}P\left(LC\right)+\xi \left(Z_{i}\right) & \\ \;\;\;\;\;\;\;\;\;\;\;\;\;\;\;\;\;\;\;\;\;\;\;\;\;\;\;\;-\left(\omega_{1}\omega_{1}^{T}P\omega_{1}\omega_{1}^{T}\right)+\omega_{1}^{T}Q_{1}\omega_{1}-\omega_{2}^{T}Q_{1}\omega_{2}+(d_{k}+1)\omega_{1}^{T}Q_{2}\omega_{1} & \\ \;\;\;\;\;\;\;\;\;\;\;\;\;\;\;\;\;\;\;\;\;\;\;\;\;\;\;\;-\omega_{3}^{T}Q_{2}\omega_{4}-\omega_{4}^{T}Q_{2}\omega_{4}\leq 0. & \end{array}$$

For (31) to be definite positive, we can write:

(32) $$\begin{array}{cc} \xi \left(Z_{i}\right)-2{\left(A+BK_{1}\right)}^{T}P\left(A+BK_{1}\right)-2{\left(BK_{2}\right)}^{T}P\left(BK_{2}\right)-2D^{T}PD+2{\left(LC\right)}^{T}P\left(LC\right) & \\ \;\;\;\;\;\;\;\;\;\;\;\;\;\;\;\;\;\;\;\;\;\;\;\;\;\;\;\;+\left(\omega_{1}\omega_{1}^{T}P\omega_{1}\omega_{1}^{T}\right)-\omega_{1}^{T}Q_{1}\omega_{1}+\omega_{2}^{T}Q_{1}\omega_{2}-\left(d_{k}+1\right)\omega_{1}^{T}Q_{2}\omega_{1} & \\ \;\;\;\;\;\;\;\;\;\;\;\;\;\;\;\;\;\;\;\;\;\;\;\;\;\;\;\;+\omega_{3}^{T}Q_{2}\omega_{3}+\omega_{4}^{T}Q_{2}\omega_{4}\geq 0\; & \end{array}$$

where: $\xi \left(Z_{i}\right)=-Z_{1}-Z_{2}-Z_{3}-Z_{4}-Z_{5}-Z_{6}$.

By putting $Z_{i}=\gamma {\bar{Z}}_{i}$, $P=\gamma {\bar{P}}^{-1},Q_{1}=\gamma {\bar{Q}}_{1}^{-1}$ and $Q_{2}=\gamma {\bar{Q}}_{2}^{-1}$, the inequality (32) can be rewritten as follows:

(33) $$\xi \left(\bar{Z}\right)-{I_{10*1}}^{T}{(B}^{T}P^{-1}B)I_{10*1}\geq 0\;$$

where: $B=\left[\begin{array}{cccccc} \sqrt{2}\left(A+BK_{1}\right) & * & * & * & * & *\\ -\omega_{1}\omega_{1}^{T} & * & * & * & * & *\\ 0_{n} & \sqrt{2}\left(BK_{2}\right) & * & * & * & *\\ 0_{n} & 0_{n} & \sqrt{2}D & * & * & *\\ 0_{n} & 0_{n} & 0_{n} & \sqrt{2}\left(LC\right) & * & *\\ 0_{n} & 0_{n} & 0_{n} & 0_{n} & \omega_{1} & *\\ 0_{n} & 0_{n} & 0_{n} & 0_{n} & -\omega_{2} & *\\ 0_{n} & 0_{n} & 0_{n} & 0_{n} & 0_{n} & \sqrt{\left(d_{k}+1\right)}\omega_{1}\\ 0_{n} & 0_{n} & 0_{n} & 0_{n} & 0_{n} & {-\omega }_{3}\\ 0_{n} & 0_{n} & 0_{n} & 0_{n} & 0_{n} & {-\omega }_{4} \end{array}\right]$
and $P^{-1}=\left[\begin{array}{ccc} {\bar{P}}_{M} & * & *\\ 0_{n} & {\bar{Q}}_{1M} & *\\ 0_{n} & 0_{n} & {\bar{Q}}_{2M} \end{array}\right]$
where: ${\bar{P}}_{M}=\left[\begin{array}{ccccc} \bar{P} & * & * & * & *\\ 0_{n} & \bar{P} & * & * & *\\ 0_{n} & 0_{n} & \bar{P} & * & *\\ 0_{n} & 0_{n} & 0_{n} & \bar{P} & *\\ 0_{n} & 0_{n} & 0_{n} & 0_{n} & \bar{P} \end{array}\right]$, ${\bar{Q}}_{1M}=\left[\begin{array}{cc} {\bar{Q}}_{1} & *\\ 0_{n} & {\bar{Q}}_{1} \end{array}\right]$ and ${\bar{Q}}_{2M}=\left[\begin{array}{ccc} {\bar{Q}}_{2} & * & *\\ 0_{n} & {\bar{Q}}_{2} & *\\ 0_{n} & 0_{n} & {\bar{Q}}_{2} \end{array}\right]$

Using Schur’s complement inequality (33) is written as:

(34) $$\left[\begin{array}{ccccccccccc} \xi \left(\bar{Z}\right) & * & * & * & * & * & * & * & * & * & *\\ \sqrt{2}\left(A+BK_{1}\right) & \bar{P} & * & * & * & * & * & * & * & * & *\\ -\omega_{1}\omega_{1}^{T} & 0_{n} & \bar{P} & * & * & * & * & * & * & * & *\\ 0_{n} & \sqrt{2}\left(BK_{2}\right) & 0_{n} & \bar{P} & * & * & * & * & * & * & *\\ 0_{n} & 0_{n} & \sqrt{2}D & 0_{n} & \bar{P} & * & * & * & * & * & *\\ 0_{n} & 0_{n} & 0_{n} & \sqrt{2}\left(LC\right) & 0_{n} & \bar{P} & * & * & * & * & *\\ 0_{n} & 0_{n} & 0_{n} & 0_{n} & \omega_{1} & 0_{n} & {\bar{Q}}_{1} & * & * & * & *\\ 0_{n} & 0_{n} & 0_{n} & 0_{n} & {-\omega }_{2} & 0_{n} & 0_{n} & {\bar{Q}}_{1} & * & * & *\\ 0_{n} & 0_{n} & 0_{n} & 0_{n} & 0_{n} & 0_{n} & 0_{n} & \sqrt{\left(d_{k}+1\right)}\omega_{1} & {\bar{Q}}_{2} & * & *\\ 0_{n} & 0_{n} & 0_{n} & 0_{n} & 0_{n} & 0_{n} & 0_{n} & {-\omega }_{3} & 0_{n} & {\bar{Q}}_{2} & *\\ 0_{n} & 0_{n} & 0_{n} & 0_{n} & 0_{n} & 0_{n} & 0_{n} & {-\omega }_{4} & 0_{n} & 0_{n} & {\bar{Q}}_{2} \end{array}\right]\geq 0$$

End of proof. □

## 4. Robust Stability of Dynamic Sensorless Active Disturbance Rejection Control Approach

In this section, robust stability analysis has been investigated for the proposed strategy. Taking into account partial input saturation and terminal constraints equality, the state observer based on the control law defined in Equation (15) has been used to robustly stabilize the closed-loop system subject to disturbances and time delay. In order to achieve that, the optimization problem is formulated, in this section, as a convex optimization problem as in [39] to construct feasibility conditions. The condition defined in Theorem 1 should guarantee asymptotic stability.

Next, in order to guarantee the feasibility of the optimization problem at each sampling time, a robust stable sensorless approach based on observer-based predictive control has been established. Consider the next optimization problem that minimizes the following worst-case quadratic objective function with an infinite horizon:

(35) $$\begin{array}{cc} \underset{u\left(k+i/k\right)}{\min}\underset{i>0}{\max}J_{\infty }(k) & \\ \mathrm{Subject}\;\mathrm{to}\;(15)\;\mathrm{and}\;{\left\|Y_{\Psi }\left(k\right)\right\|}_{2}\leq y_{max} & \end{array}$$

where $\underset{i>0}{\max}J_{\infty }(k)$ is the upper bound γ, that is, the objective target to optimize, which is related to the following objective function as follows next:

(36) $$J_{\infty }\left(k\right)=\sum_{i=0}^{\infty }\left[{\left\|\tilde{e}\left(k+i\right)\right\|}_{Q_{0}}^{2}+{\left\|u\left(k+i\right)\right\|}_{R_{0}}^{2}\right].$$

Moreover, let us define the estimation error dynamics as follows:

(37) $$e\left(k+1\right)=Ae\left(k\right)+De_{E}\left(k\right)-LCe\left(k\right).$$

**Theorem** **2.** *Given the augmented system (37) that reflected the discrete-time Markovian switching systems (10). The state feedback controller based on the dynamic system in (17) meets the control performances (15), and will asymptotically stabilize system (5) subject to disturbance, time-delay and partial input saturation, if there exists a positive scalar* γ *, positive definite matrices* $P,\;X_{1}\;and\;X_{2}$ *, and any matrices* $K_{1},\;K_{2}$ *and* L *satisfying the following LMIs:*

(38) $$\underset{\gamma ,Q,X_{1},X_{2},K_{1},\;K_{2},L}{\min}\gamma \;$$

*Subject to*

(39) $$\left[\begin{array}{cc} 1 & *\\ e\left(k\right) & Q \end{array}\right]\geq 0\;$$

(40) $$\left[\begin{array}{cc} y_{max}^{2} & C\left(AG+D{℘}_{e}G-LCG\;\right)\\ {\left(AG+D{℘}_{e}G-LCG\;\right)}^{T}C^{T} & Q-G^{T}-G \end{array}\right]\geq 0\;$$

(41) $$\left[\begin{array}{cccccccc} Q-X_{1} & * & * & * & * & * & * & *\\ R_{0}^{1/2}(\omega_{3}BK_{1}+\omega_{4}BK_{2}) & -\gamma I & * & * & * & * & * & *\\ Q_{0}^{1/2}\omega_{1} & 0_{n} & -\gamma I & * & * & * & * & *\\ \sqrt{\left(d_{k}+1\right)}\omega_{1} & 0_{n} & 0_{n} & {-X}_{2} & * & * & * & *\\ \left(A+D{℘}_{e}-LC\right) & 0_{n} & 0_{n} & 0_{n} & -Q & * & * & *\\ \omega_{2} & 0_{n} & 0_{n} & 0_{n} & 0_{n} & X_{1} & * & *\\ \omega_{3} & 0_{n} & 0_{n} & 0_{n} & 0_{n} & 0_{n} & X_{2} & *\\ \omega_{4} & 0_{n} & 0_{n} & 0_{n} & 0_{n} & 0_{n} & 0_{n} & X_{2} \end{array}\right]\geq 0$$

**Proof of Theorem** **2.** Taking the forward difference $∆V\left(k\right)$ of (19) with upper bound γ, and for any i≥0, with respect to time along the trajectory of the system. The objective is to satisfy the following stability constraint as follows:

(42) $$∆V\left(k\right)=V\left(k+1\right)-V\left(k\right)\leq \gamma \;$$

(43) $$V\left(e\left(k+i+1/k\right)\right)-V\left(e\left(k+1/k\right)\right)\leq -\left[{\left\|\tilde{e}\left(k+i\right)\right\|}_{Q_{0}}^{2}+{\left\|u\left(k+i\right)\right\|}_{R_{0}}^{2}\right].$$

To construct the stability conditions given in Theorem 2, the summation is performed up to ∞, i.e., i→∞, e(i|k) should approach zero, i.e., $\hat{\Psi }\left(\infty |k\right)=0$, yields:

(44) $$J\left(k\right)\leq V\left(e\left(k/k\right)\right)\leq \gamma .$$

Obviously, for asymptotical stability of DC-DC converters inequality (41) implies that $V\left(e\left(k/k\right)\right)$ decreases as i→∞ and (48) is formulated as follows next:

(45) $$e^{T}\left(k\right)Pe\left(k\right)\leq \gamma .$$

By using the Schur complement, the LMI (39) is obtained.

- While the output constraint in Equation (13) is expressed as:

(46) $${\left\|Y_{\Psi }\left(k+i/k\right)\right\|}_{max}≜\underset{i}{\max}Y_{\Psi i}\left(k+i/k\right).$$

It can be written as: ${\left\|Y_{\Psi }\left(k\right)\right\|}_{2}\leq y_{max}^{2}$

(47) $$\underset{i>0}{\max}{\left\|Y_{\Psi }\left(k\right)\right\|}_{max}\geq \underset{i>0}{\max}{\left\|C\left(Ae\left(k\right)+De_{E}\left(k\right)-LCe\left(k\right)\;\right)\right\|}_{max}.$$

Thus, ${\left\|Y_{\Psi }\left(k+i/k\right)\right\|}_{2}\leq y_{max},\;i\geq 1$, for any $\left[A\left(k+j\right)\;B\left(k+j\right)\;D\left(k+j\right)\right]\in \Omega ,j\geq 1$ if:

(48) $$P^{1/2}\left[{\left(A+D{℘}_{e}-LC\;\right)}^{T}C^{T}C\left(A+D{℘}_{e}-LC\;\right)\right]P^{\frac{1}{2}}\leq y_{max}^{2}I.\;$$

Using the Schur complement the output constraint can write as follows:

(49) $$\left[\begin{array}{cc} y_{max}^{2} & C\left(A+D{℘}_{e}-LC\;\right)\\ {\left(A+D{℘}_{e}-LC\;\right)}^{T}C^{T} & -P^{-1} \end{array}\right]\geq 0$$

Multiplying the right by $\left[\begin{array}{cc} I & O\\ 0 & G \end{array}\right]$ and the left by $\left[\begin{array}{cc} I & O\\ 0 & G^{T} \end{array}\right]$, the LMI (40) is obtained.

- Let us recall the optimization problem represented in expression (43) that defines $∆V\left(k/k\right)$, and we have:

(50) $$V\left(e\left(k+i+1/k\right)\right)-V\left(e\left(k+1/k\right)\right)\leq -\left[{\left\|\tilde{e}\left(k+i\right)\right\|}_{Q_{0}}^{2}+{\left\|u\left(k+i\right)\right\|}_{R_{0}}^{2}\right].$$

According to (14) and (37), the optimization problem can be written as:

(51) $$\begin{array}{cc} {\left(Ae\left(k\right)+De_{E}\left(k\right)-LCe\left(k\right)\right)}^{T}P\left(Ae\left(k\right)+De_{E}\left(k\right)-LCe\left(k\right)\right)-\left(e^{T}\left(k/k\right)Pe(k/k)\right) & \\ \;\;\;\;\;\;\;\;\;\;\;\;\;\;\;\;\;\;\;\;\;\;\;\;\;\;\;\;+\sum_{j=k-d_{k}}^{k-1}{\tilde{e}}^{T}\left(j\right)Q_{1}\tilde{e}\left(j\right)+\sum_{j=1-d_{M}}^{1-d_{m}}\sum_{r=k-1-j}^{k-1}{\tilde{e}}^{T}\left(r\right)Q_{2}\tilde{e}\left(r\right) & \\ \;\;\;\;\;\;\;\;\;\;\;\;\;\;\;\;\;\;\;\;\;\;\;\;\;\;\;\;\leq -{\tilde{e}}^{T}\left(k\right)Q_{0}\tilde{e}\left(k\right)-u^{T}\left(k\right)R_{0}u\left(k\right)\; & \end{array}$$

where $\tilde{e}={\left[\begin{array}{cccc} e(k/k) & e_{E}(k/k) & \hat{\Psi }(k/k) & \hat{\Psi }(k-d/k) \end{array}\right]}^{T}$.

Then, it can be written:

(52) $$\begin{array}{cc} {\left(A+D{℘}_{e}-LC\right)}^{T}{\tilde{e}}^{T}\left(k\right)P\tilde{e}\left(k\right)\left(A+D{℘}_{e}-LC\right)-\left(\omega_{1}{\tilde{e}}^{T}\left(k\right)P\tilde{e}\left(k\right){\omega_{1}}^{T}\right)+{\tilde{e}}^{T}\left(k\right)Q_{1}\tilde{e}\left(k\right) & \\ \;\;\;\;\;\;\;\;\;\;\;\;\;\;\;\;\;\;\;\;\;\;\;\;\;\;\;\;-{\tilde{e}}^{T}\left(k-d_{k}\right)Q_{1}\tilde{e}\left(k-d_{k}\right)+{\left(d_{k}+1\right)\tilde{e}}^{T}\left(k\right)Q_{2}\tilde{e}\left(k\right) & \\ \;\;\;\;\;\;\;\;\;\;\;\;\;\;\;\;\;\;\;\;\;\;\;\;\;\;\;\;-{\tilde{e}}^{T}\left(k-d_{M}\right)Q_{2}\tilde{e}\left(k-d_{M}\right)-{\tilde{e}}^{T}\left(k-d_{m}\right)Q_{2}\tilde{e}\left(k-d_{m}\right)+{\tilde{e}}^{T}\left(k\right)Q_{0}\tilde{e}\left(k\right) & \\ \;\;\;\;\;\;\;\;\;\;\;\;\;\;\;\;\;\;\;\;\;\;\;\;\;\;\;\;+u^{T}\left(k\right)R_{0}u\left(k\right)\leq 0\; & \end{array}$$

where:

(53) $$\xi \left(k\right)={\left[\begin{array}{ccc} {\tilde{e}}^{T}\left(k\right) & {\tilde{e}}^{T}\left(k-d_{k}\right) & \begin{array}{cc} {\tilde{e}}^{T}\left(k-d_{M}\right) & {\tilde{e}}^{T}\left(k-d_{m}\right) \end{array} \end{array}\right]}^{T}$$

(54) $$\begin{array}{cc} \omega_{1}^{T}{\left(A+D{℘}_{e}-LC\right)}^{T}P\omega_{1}\left(A+D{℘}_{e}-LC\right)-\omega_{1}^{T}P\omega_{1}+\omega_{1}^{T}Q_{1}\omega_{1}-\omega_{2}^{T}Q_{1}\omega_{2} & \\ \;\;\;\;\;\;\;\;\;\;\;\;\;\;\;\;\;\;\;\;\;\;\;\;\;\;\;\;+\left(d_{k}+1\right)\omega_{1}^{T}Q_{2}\omega_{1}-\omega_{3}^{T}Q_{2}\omega_{3}-\omega_{4}^{T}Q_{2}\omega_{4}+\omega_{1}^{T}Q_{0}\omega_{1} & \\ \;\;\;\;\;\;\;\;\;\;\;\;\;\;\;\;\;\;\;\;\;\;\;\;\;\;\;\;+{\left(\omega_{3}BK_{1}+\omega_{4}BK_{2}\right)}^{T}R_{0}(\omega_{3}BK_{1}+\omega_{4}BK_{2})\leq 0. & \end{array}$$

Then, it can be written as:

(55) $$\left[\begin{array}{cc} \Lambda & *\\ 0_{n} & \Xi \end{array}\right]-{\left[\begin{array}{c} \left(A+D{℘}_{e}-LC\right)\\ \omega_{2}\\ \omega_{3}\\ \omega_{4} \end{array}\right]}^{T}\left[\begin{array}{cccc} -P & * & * & *\\ 0_{n} & Q_{1} & * & *\\ 0_{n} & 0_{n} & Q_{2} & *\\ 0_{n} & 0_{n} & 0_{n} & Q_{2} \end{array}\right]\left[\begin{array}{c} \left(A+D{℘}_{e}-LC\right)\\ \omega_{2}\\ \omega_{3}\\ \omega_{4} \end{array}\right]\geq 0.$$

By putting: $\Lambda =P-Q_{1}-\omega_{1}^{T}Q_{0}\omega_{1}-{\left(\omega_{3}BK_{1}+\omega_{4}BK_{2}\right)}^{T}R_{0}(\omega_{3}BK_{1}+\omega_{4}BK_{2})$ and $\Xi =-\left(d_{k}+1\right)\omega_{1}^{T}Q_{2}\omega_{1}$.

By using the Schur complement, it obtains:

(56) $$\left[\begin{array}{cccccc} \Lambda & * & * & * & * & *\\ 0_{n} & \Xi & * & * & * & *\\ \left(A+D{℘}_{e}-LC\right) & 0_{n} & -P & * & * & *\\ \omega_{2} & 0_{n} & 0_{n} & Q_{1} & * & *\\ \omega_{3} & 0_{n} & 0_{n} & 0_{n} & Q_{2} & *\\ \omega_{4} & 0_{n} & 0_{n} & 0_{n} & 0_{n} & Q_{2} \end{array}\right]\geq 0.$$

Substituting $P=\gamma Q^{-1}$, $Q_{1}=\gamma X_{1}^{-1}$ and $Q_{2}=\gamma X_{2}^{-1}$ into (56) and using t Schur complement, (56) can be expressed as:

(57) $$\left[\begin{array}{cccccccc} Q-X_{1} & * & * & * & * & * & * & *\\ R_{0}^{1/2}(\omega_{3}BK_{1}+\omega_{4}BK_{2}) & -\gamma I & * & * & * & * & * & *\\ Q_{0}^{1/2}\omega_{1} & 0_{n} & -\gamma I & * & * & * & * & *\\ \sqrt{\left(d_{k}+1\right)}\omega_{1} & 0_{n} & 0_{n} & {-X}_{2} & * & * & * & *\\ \left(A+D{℘}_{e}-LC\right) & 0_{n} & 0_{n} & 0_{n} & -Q & * & * & *\\ \omega_{2} & 0_{n} & 0_{n} & 0_{n} & 0_{n} & X_{1} & * & *\\ \omega_{3} & 0_{n} & 0_{n} & 0_{n} & 0_{n} & 0_{n} & X_{2} & *\\ \omega_{4} & 0_{n} & 0_{n} & 0_{n} & 0_{n} & 0_{n} & 0_{n} & X_{2} \end{array}\right]\geq 0.$$

End of proof. □

To show the best visibility of the contribution proposed in this paper and prove the effectiveness of the dynamic sensorless active disturbance rejection-based predictive control approach, Table 1 presents a performance comparison with similar and related works in the literature.

## 5. Simulation Results and Discussions

In this section, the simulation results with discussions are given to illustrate the effectiveness and improvement of the proposed control approach. The disturbances rejection and compensation of time delay are tested to achieve the dynamic robustness of the PWM DC-DC converter. The simulation results are presented in two scenarios: the buck mode and buck/boost mode. For the first mode desired, the value as constraints are fixed, the closed loop DC-DC converter is targeted for 15 V/1.25 A, while the buck/boost mode is fixed to achieve the target of the next sequence, $\left[15-23-10\right]\;V/\left[1.25-2.5-0.8\right]\;A,$ according to the duty cycle d(t). Then, a comparison of the obtained results, with two strategies, is discussed in detail. The two strategies used for comparison are: dynamic sensorless active disturbances rejection-based predictive control and the second strategy is the Robust Classical MPC.

The PWM DC-DC converter is subject to load variation such as disturbances, time delay, and partial input saturation. These variations may influence the output voltage/current tracking control. To deal with these parameters to maintain the desired output values of the DC-DC converters, a controller, as discussed earlier, is designed from the convex LMI optimization problem.

The parameters of the PWM DC-DC converter are defined in Table 2.

The selected MPC parameters as weighting matrices are $Q_{0}=I_{n},R_{0}=0.5$; the constraints in the buck mode are 15V/1.25A and in the buck/boost mode are $\left[15-23-10\right]V/\left[1.25-2.5-0.8\right]$. The initials conditions are $x={\left[0\;0\right]}^{T}$.

- ***Case 1. PWM Buck DC-DC Converter***

**Figure 1:**
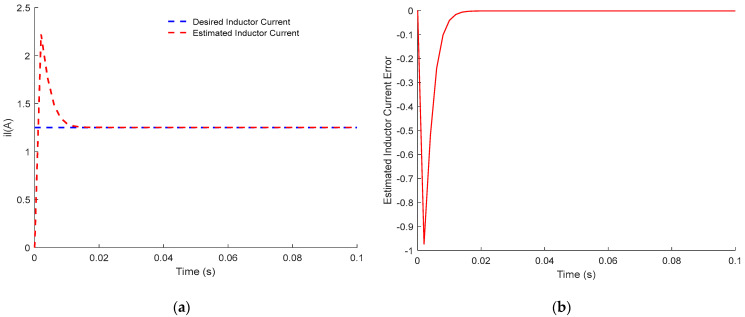
Responses of the inductance current of PWM buck converter: (**a**) Inductor Current Tracking Response; (**b**) Inductor Current Tracking Error.

**Figure 2:**
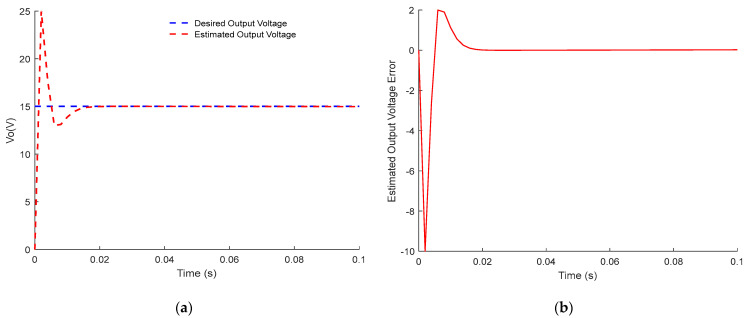
Responses of the output voltage of PWM buck converter: (**a**) Output Voltage Tracking Response; (**b**) Output Voltage Tracking Error.

Both responses of the output voltage and the inductance current of the PWM buck converter are, respectively, plotted in Figure 1 and Figure 2.

Figure 1 and Figure 2 show up the estimated inductor current and its error dynamics, and the estimated output voltage and its error dynamics, respectively. For both results, the dynamic sensorless active disturbance rejection-based predictive control tracks the desired trajectory precisely at 15 V/1.25 A (voltage/current trajectory) at t = 0.02 s. In addition, the results obtained from the proposed controller show consistent dynamical response, compensation of time delay, and disturbance rejection capability.

- ***Comparison with Classical Robust MPC***

**Figure 3:**
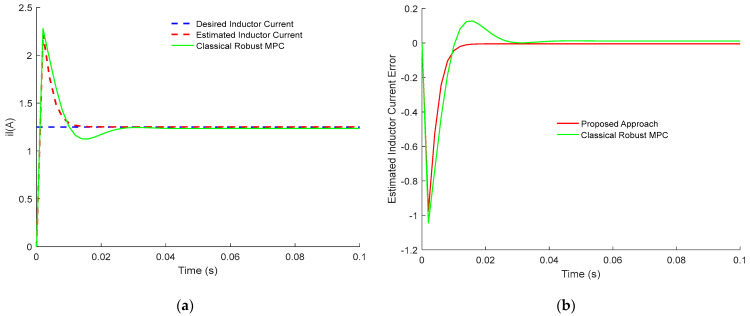
Comparison between the tracking response of the proposed control design and classical robust MPC for PWM buck DC-DC converter: (**a**) Inductor Current Tracking Response; (**b**) Inductor Current Tracking Error.

**Figure 4:**
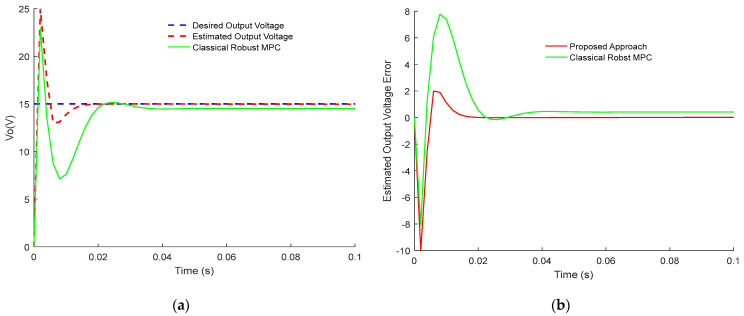
Comparison between the tracking response of the proposed control design and classical robust MPC, for PWM buck DC-DC converter: (**a**) Output Voltage Tracking Response; (**b**) Output Voltage Tracking Error.

Figure 3 and Figure 4 demonstrate the comparison results between the presented control approach and the classical robust MPC of Buck PWM DC-DC power converter. Notably, the two designs track the desired value subject to disturbances and time delay. However, the proposed robust sensorless active disturbances rejection predictive control strategy has a faster dynamical response and perfect disturbance rejection compared to the classical approach. In addition, the proposed approach has better compensation for the time-dealy than the classical approach. Clearly, the estimated error dynamics demonstrate the tracking dynamic of inductor current and output voltage response. Thus, the terminal constraints are satisfied using dynamic sensorless active disturbance rejection-based predictive control while it is not the case for the classical robust MPC. Moreover, the settling time for the proposed approach is less than 15 ms, while in classical robust MPC, it is more than 25 ms. Furthermore, it is clear from Figure 3 and Figure 4 that tracking dynamics using classical robust MPC will not achieve the desired targets.

When investigating the effectiveness of each control approach, it is well noticed that the sensorless active disturbance rejection-based predictive control strategy has more abilities in rejecting the disturbances. In addition, the proposed approach has the ability to compensate for time delay compared to the classical robust MPC.

- ***Case 2. PWM Buck/Boost DC-DC Converter***

In this subsection, two cycles are considered to investigate the dynamic response. The first is increasing the desired value (voltage/current) from 15 V to 23 V/1.25 A to 2.5 A, respectively, and the second cycle is when the line value decreases from 23 V to 10 V/2.5 A to 0.8 A, respectively.

**Figure 5:**
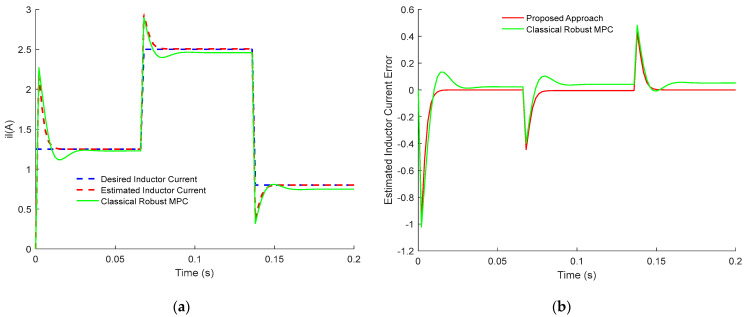
Responses of the output voltage of PWM buck/boost DC-DC converter: (**a**) Inductor Current Tracking Response; (**b**) Inductor Current Tracking Error.

**Figure 6:**
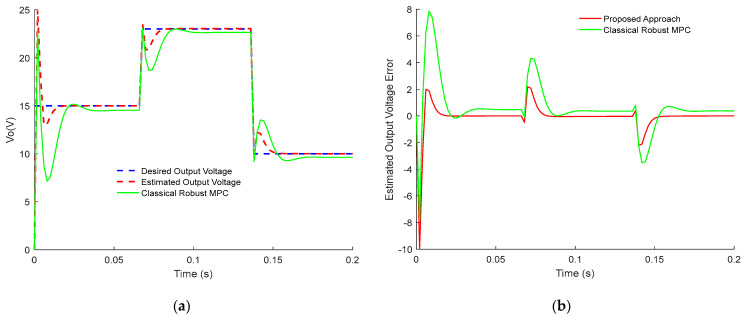
Responses of the output voltage of PWM buck/boost DC-DC converter: (**a**) Output Voltage Tracking Response; (**b**) Output Voltage Tracking Error.

Figure 5 and Figure 6 show the tracking performances for dynamic sensorless active disturbance rejection-based predictive control and the classical robust MPC strategies as buck/boost mode with switching mode at a specific time according to the duty cycle d(t). In Figure 5, the settling time for the proposed approach occurs in less than 1 ms at the first switching, from 15 V to 23 V/1.25 A to 2.5 A, while in classical robust MPC is more than 2 ms. For the second cycle from 23 V to 10 V/2.5 A to 0.8 A, the regulation time is occurring in less than 1 ms and more than 2 ms for the proposed approach and classical robust MPC, respectively. It can be noticed that dynamic sensorless active disturbance rejection-based predictive control exhibit a percentage overshoot/undershoot less than those exhibited by the Robust Classical MPC. In addition, the proposed approach achieves the desired target, deals with disturbances, compensates for time delay, and satisfies the constraints. On the contrary, the estimated error dynamic in Figure 5 and Figure 6 has proved that classical robust MPC has not been able to efficiently track precisely the desired values. In addition, the classical MPC approach has demonstrated less efficiency in dealing with disturbances and compensation for time delay.

In conclusion, the obtained results in Figure 1, Figure 2, Figure 3, Figure 4, Figure 5 and Figure 6 show the superiority and effectiveness of the proposed control strategy in satisfying the imposed constraints, dealing with disturbances, and compensating for the time delay in the presence of partial input saturation.

## 6. Conclusions

In this work, a dynamic sensorless active disturbances rejection control approach is proposed and applied to PWM DC-DC converters. Taking into account partial input saturation and terminal equality constraints of the DC-DC switching power converters, based on observer-based predictive control, less conservative conditions are established via Linear Matrix Inequalities. The main challenge presented in this work is converting the high nonlinearity of DC-DC converters into linear systems with modeling uncertainties; meanwhile, handling load variations as external disturbances that had been rejected to maintain robust performances of the PWM DC-DC converters. In addition, time delay had considered in the input control as an extra parameter. Therefore, the Lyapunov–Krasovskii function had used to construct the required necessary and less conservative stability conditions to ensure the robust performance of the system. These conditions can be obtained by using the infinite time domain “min–max”, to formulate the optimization problem into a new convex optimization problem. As a result, the proposed sensorless active disturbances rejection control strategy based on the observer–predictive control is designed, in which the parameters of the control law are updated at each iteration to obtain the control signal; meanwhile, the asymptotical stability of the PWM DC-DC power converter is guaranteed. The simulation results of the two scenarios have shown effectiveness and the reliability of the proposed approach. So, disturbances rejection is ensured and time delay had compensated, with satisfying the imposed constraints.

## Figures and Tables

**Table 1:** Performances comparison of recent control approaches of a DC-DC converter.

Control Strategy	Used Technics	Advantages	Reference
Dynamic Sensorless Active Disturbances Rejection-based Predictive Control	- Predictive Control	- Fast-tracking performances	Present Work
		- Robustness and stability	
	- Sensorless Control-based Observer (Voltage/Current)	- Accurate tracking	
		- Chattering alleviation	
	- Disturbance Observer	- Time-varying load estimation	
		- Less conservative conditions	
Sensorless Predictive Control	- Predictive Control	- Fast-tracking performances	[37]
	- Voltage Sensorless Control based Observer	- Robustness and stability	
		- Fast response	
State Observe-based Control	- Sensorless Control-based Observer	- Fast response	[30]
		- Time-varying load estimation	
Robust PWM-based Sliding Mode Control	- Sliding Mode Control	- Fast response	[40]
	- Disturbance Observer	- Robustness	
Finite-time Output Feedback Sensorless Control	- Finite-time Output Feedback Control	- Fast response	[29]
	- State Observer	- Stability	
	- Current Sensorless Control	- Decreasing of chattering	
Sensorless Control	- Voltage Sensorless Control	- Fast response	[31]
	- PI Controller	- Stability	
	- State Observer		
Robust Nonlinear Current-Mode Control	- Sliding-mode Current Control	- Tracking performances	[41]
		- Stability	

**Table 2:** DC-DC converter physical parameters.

Parameters	Description	Numerical Value
*V_in_*	Input voltage	25 V
*V_Ref_*	Desired voltage	15 V–23 V
I_lmin_, I_lmax_	Desired range current	0.5 A, 3 A
*i_load_*	External disturbance	0.25sin(1000 t)
*R*	The load resistance	6 Ω
*L*	The inductance	98.58 mH
*R_l_*	Resistance of inductor	48.5 mΩ
*C*	The capacitance	202.5 µF
*R_c_*	Resistance of capacitor	0.16 mΩ
*R_m_*	On-state resistance of the MOSFET	0.27 mΩ
